# Bayesian Analysis of Finite Populations under Simple Random Sampling

**DOI:** 10.3390/e23030318

**Published:** 2021-03-08

**Authors:** Manuel Mendoza, Alberto Contreras-Cristán, Eduardo Gutiérrez-Peña

**Affiliations:** 1 Departamento de Estadística, Instituto Tecnológico Autónomo de México, Río Hondo 1, Ciudad de México 01080, Mexico; 2 Departamento de Probabilidad y Estadística, Instituto de Investigaciones en Matemáticas Aplicadas y en Sistemas, Universidad Nacional Autónoma de México, Apartado Postal 20-126, Ciudad de México 01000, Mexico; alberto@sigma.iimas.unam.mx (A.C.-C.); eduardo@sigma.iimas.unam.mx (E.G.-P.)

**Keywords:** survey sampling, superpopulation, predictive analysis, nonparametric modeling

## Abstract

Statistical methods to produce inferences based on samples from finite populations have been available for at least 70 years. Topics such as *Survey Sampling* and *Sampling Theory* have become part of the mainstream of the statistical methodology. A wide variety of sampling schemes as well as estimators are now part of the statistical folklore. On the other hand, while the Bayesian approach is now a well-established paradigm with implications in almost every field of the statistical arena, there does not seem to exist a conventional procedure—able to deal with both continuous and discrete variables—that can be used as a kind of default for Bayesian survey sampling, even in the simple random sampling case. In this paper, the Bayesian analysis of samples from finite populations is discussed, its relationship with the notion of superpopulation is reviewed, and a nonparametric approach is proposed. Our proposal can produce inferences for population quantiles and similar quantities of interest in the same way as for population means and totals. Moreover, it can provide results relatively quickly, which may prove crucial in certain contexts such as the analysis of quick counts in electoral settings.

## 1. Introduction

Survey sampling is one of the most popular areas of Applied Statistics. Neyman (1934) [1] established the methodological foundations for statistical inference based on random samples obtained from finite populations. This approach, known as *design-based* inference, has become a standard mostly due to its early adoption by international agencies for gathering and analyzing data from different countries.

The general spirit of these methods is nonparametric as no assumptions are made regarding the distribution of the variable of interest. Given a population of size *N* and a variable of interest *X*, inferences focus on some attributes of the set $X=\{X_{1},\ldots ,X_{N}\}$. Special interest is given to the population total, $T=\sum_{i=1}^{N}X_{i}$, and the population mean, ${\bar{X}}_{\left(N\right)}=T/N$. For the simpler designs, point-wise estimates based on a sample of size *n*, $\left\{x_{i_{1}},\ldots ,x_{i_{n}}\right\}$, are least squares optimal and are often unbiased. Here, $\left\{i_{1},\ldots ,i_{n}\right\}\subset \left\{1,\ldots ,N\right\}$ denotes the set of indices corresponding to the *n* sampled units within the population. Interval estimation relies on the asymptotic normality of the corresponding sampling distribution.

These techniques are widely applicable and extraordinarily useful. On the other hand, however, the results are not necessarily optimal in a general statistical sense (see Godambe (1955) [2] for an early discussion on this topic) and inferences might fail if asymptotic normality does not hold.

The application of Bayesian ideas to survey sampling has been explored for some time now, and falls into the realm of *model-based inference* (Little (2004) [3]). Pearson (1928) [4] applied inverse probability theory to produce inferences on the proportion of the elements of a finite population satisfying a given condition. His assumptions that the population is finite and that the sampling is without replacement lead to a unique, well-defined model, namely, hypergeometric; thus, his analysis is fully parametric. Pearson produced a posterior distribution for the unknown proportion under the assumption of a uniform prior and showed that, in general, it is not compatible with the frequentist results based on asymptotic normality.

Aggarwal (1959) [5] formulated the estimation of the mean of a finite population as a decision problem and proved that under both simple random sampling and stratified sampling, point-wise classical results can be recovered as a limit of the Bayesian solution when a quadratic loss function is used and the prior variance goes to infinity. In a similar fashion, Godambe (1965) [6] discussed a class of admissible classical estimates and proved in passing that they can also be seen as Bayesian estimates, whereas Aggarwal (1966) [7] generalized his previous results to the case of two-stage sampling.

On the other hand, Godambe (1966) [8], using a quadratic loss function, found a Bayesian estimate for the population total which depends on the prior distribution only through the predictive expected value of *X* for the nonsampled units. Specifically, if a sample of size *n* is obtained whose elements have indexes in a set S⊂{1,…,N}, then $\hat{T}=\sum_{i\in S}x_{i}+\sum_{i\notin S}E\left(X_{i}\right)$, where E(X) stands for the corresponding prior predictive mean.

Ericson (1969) [9] assumed X are obtained from a sequence of exchangeable random variables; therefore, their joint distribution can be represented as $p\left(x\right)=\int \prod_{i=1}^{N}p\left(x_{i}|\theta \right)p\left(\theta \right)d\theta$. This representation motivated the idea of an infinite superpopulation from which X can be considered a set of i.i.d. observations. The superpopulation is described through the parametric model p(x|θ), and the remaining uncertainty regarding the parameter θ is taken into account via a prior p(θ). If the selection probability for each unit does not depend on the value of *X* (a noninformative sampling scheme), the sample $\left\{x_{i_{1}},\ldots ,x_{i_{n}}\right\}$ can also be regarded as a set of i.i.d. observations from the superpopulation.

This framework provides a setting where inferences on the population total $T=\sum_{i\in S}x_{i}+\sum_{i\notin S}X_{i}$ can be obtained by noting that, upon observing the sample, $T_{obs}=\sum_{i\in S}x_{i}$ is a known constant, whereas $T_{nobs}=\sum_{i\notin S}X_{i}$ can be described through its corresponding posterior predictive distribution. Ericson derived the results for the case of a continuous variable, assuming a normal distribution for the superpopulation. He proved that the asymptotic classical results can be obtained as the prior becomes noninformative. His approach can be replicated with any other model p(x|θ), although he recognized that adoption of a specific model for the superpopulation is a restrictive assumption. In the case where the support of *X* is finite and completely known, he proposed a multinomial model for the superpopulation and, via a (Dirichlet) conjugate analysis, obtained a robust solution which recovers Pearson’s results for the binary case. This approach, however, is not general as it requires the support of *X* to be known and finite.

In the case of complex survey designs, both parametric and nonparametric Bayesian approaches have been proposed. Fox example, Si et al. (2015) [10] use a hierarchical approach in which they model the distribution of the weights of the nonsampled units in the population and simultaneously include them as predictors in a nonparametric Gaussian-process regression model. More recently, Rahnamay Kordasiabi and Khazaei (2020) [11] study a Bayesian nonparametric model, based on a Dirichlet process prior, that considers the inverse-probability weights as the only available information. Their model is essentially a Bayesian nonparametric mixture of regression models for the survey outcomes with the weights as predictors. Unfortunately, as in the frequentist approach, there are several ways of incorporating the inclusion probabilities into the analysis, and it is not always clear which of the available methods is the most adequate in a given situation, see, for example, Gelman (2007) [12]. Si, et al. (2020) [13] combine Bayesian prediction and weighting to provide a unified approach to survey inference.

The above notwithstanding, even in the simple random sampling case there does not seem to exist a conventional procedure that can be used as a kind of default for Bayesian survey sampling. In this paper, we discuss a flexible Bayesian nonparametric approach to the analysis of samples from finite populations in the context of simple random sampling, which can be easily generalized to the case of stratified sampling schemes. Our proposal can deal with both continuous and discrete variables, and can produce inferences for population quantiles and similar quantities of interest in the same way as for population means and totals. Moreover, it can provide results relatively quickly, which may prove crucial in certain contexts such as the analysis of quick counts.

The layout of the paper is as follows. In the next section, we make the case for the use of nonparametric, robust models. We then present our proposal in Section 3. Details of the implementation as well as some illustrative examples concerning some basic sampling schemes are given in Section 4. Finally, Section 5 contains some concluding remarks.

## 2. The Search for Robustness

Several authors have followed the Bayesian approach to analyze complex survey designs using specific superpopulation models (see Royall and Pfeffermann (1982) [14] and Treder and Sedransk (1996) [15], for example). Other proposals have tried to robustify the analysis with respect to the choice of the superpopulation distribution. Binder (1982) [16] replaced the Dirichlet prior distribution of Ericson with a Dirichlet prior process and removed the requirement on the support of *X*. However, he only offered an asymptotic approximation to the complete posterior distribution of *T*, which happens to be normal. The results coincide with their classical counterpart.

Lo (1988) [17] proposed a bootstrap mechanism, based on a Pólya urn scheme, to simulate copies of the entire population from which estimates of the finite population distribution and of some parameters of interest can be obtained. The underlying distribution is a Dirichlet-multinomial process, which can be interpreted as an approximation to a posterior distribution when a “noninformative” prior process is adopted. In that paper, the posterior expected value for the population mean was calculated and shown to coincide with the classical estimator. Conditions for asymptotic normality are established and the classical interval estimates are also reproduced.

In Lazar, et al. (2008) [18], the idea of generating “copies” of the entire finite population using the Pólya urn for a fixed sample size was pursued. There, the main interest was no longer on asymptotic results. Instead, the population parameters were computed for each copy, and thus the relevant posterior distribution was approximated via simulation. Point-wise as well as interval estimates can then be computed for any sample size. This procedure involves, however, a discrete predictive distribution whose support is restricted to that of the observed sample. To get around this constraint, Martínez-Ovando, et al. (2014) [19] proposed a nonparametric approach which allows one to incorporate any prior information available. The uncertainty about the population distribution of the (continuous) variable of interest was described through a species sampling model. The authors obtained an expression for the marginal predictive distribution of each unsampled unit and exhibited the posterior mean of the population total. They also argued that the complete posterior distribution for the population total can be obtained via a convolution of the individual predictive distributions. They illustrated their approach with simple random sampling, as well as with strata and unplanned domains. This approach is somewhat limited as the underlying variable is assumed to be continuous. This is a major issue, as in many applications the variable of interest is discrete.

More recently, as pointed out in the previous section, other authors have dealt with complex survey designs from a nonparametric Bayesian perspective. In that setting, one of the main issues is how to take into account the sampling weights of the design. Among other ideas, some authors have used the weights to build a pseudo-likelihood function (see Savitsky and Toth (2016) [20], for example), whereas others have fitted a regression model as part of the inference process (Rahnamay Kordasiabi and Khazaei (2020) [11]).

Our approach is based on a Bayesian nonparametric Dirichlet process mixture (DPM) model. Such models are extremely flexible; a large class of population distributions can in fact be consistently estimated using this kind of model (Ghosal, et al. (1999) [21]; Ghosh and Ramamoorthi (2003) [22]). Specifically, if the true density is in the Kullback–Leibler support of the DPM prior, then the corresponding posterior is consistent.

## 3. Our Proposal

From a general perspective, inferences on the population total, as well as on any other population parameter Q(X), can be obtained by recalling that *Q* is a function of the complete set of values $X=\{X_{1},\ldots ,X_{N}\}$, which can then be regarded as a set of unknown parameters. Thus, a prior distribution must then be chosen to describe the knowledge concerning X, and the sample must be used to update this knowledge and get the corresponding posterior distribution. Once this distribution is available, the posterior for *Q* must be obtained. The exchangeability assumption for $X=\{X_{1},\ldots ,X_{N}\}$ leads to the use of a (parametric) model p(x|θ) for the population distribution. In this setting, the random variables $X_{1},\ldots ,X_{N}$ are conditionally i.i.d. given θ according to this model, which would correspond to the superpopulation distribution.

As discussed above, this parametric structure may be simple and the analysis leading to the predictive distribution of Q(X) may be straightforward, but it has also been shown to be rather restrictive as a proposal for a general Bayesian survey sampling methodology as it depends on the specific superpopulation model that is used (see Sedransk (2008) [23], for example). Some authors have relaxed the parametric assumption and assumed instead that the population distribution belongs to some nonparametric family, see, for example, Rahnamay Kordasiabi and Khazaei (2020) [11], who assume that the underlying variable of interest is continuous.

Moving to a nonparametric framework, let us assume that $X_{j}|\theta_{j}∼p\left(x|\theta_{j}\right),$ where $\left\{\theta_{j}\right\}$ are conditionally independent and such that $\theta_{j}|G∼G$. A widely used nonparametric family of prior distributions for *G* is that corresponding to the Dirichlet Process (Ferguson (1973) [24]). If a random probability measure *G* follows a Dirichlet Process with precision parameter α and base probability measure $G_{0}$—denoted $G∼DP(\alpha ,G_{0})$—then, according to Sethuraman (1994) [25], it can be represented as

(1) $$G\left(\cdot \right)=\sum_{j=1}^{\infty }\omega_{j}\delta_{\theta_{j}}\left(\cdot \right),$$

where $\theta_{1},\theta_{2},\ldots$ are independent and with distribution $G_{0},$ and the weights ${\left\{\omega_{j}\right\}}_{j}$ are obtained from a sequence $\left\{\upsilon_{j}\right\}$ of independent and identically distributed Beta(1,α) random variables by setting $\omega_{1}=\upsilon_{1}$ and $\omega_{sj}=\upsilon_{j}\prod_{l<j}\left(1-\upsilon_{l}\right),$ for j=2,3,…. In the stick breaking representation (1), $\delta_{\theta }$ denotes the measure with a point mass of 1 at θ.

Adopting a Dirichlet Process as the prior distribution for *G* leads us to the DPM model for the superpopulation distribution:

f(x|G)=∫p(x|θ)dG(θ).

Note that, using (1), we can write

(2) $$f\left(x|G\right)=\sum_{j=1}^{\infty }\omega_{j}p\left(x|\theta_{j}\right).$$

Walker (2007) [26] provides a slice sampler MCMC algorithm which uses latent variables to produce posterior samples from (2). In this sampling scheme, the assumption that the random measure *G* has been integrated out is not required, and therefore the Pólya Urn scheme is not used. Kalli, et al. (2011) [27] suggest some efficiency improvements to the algorithm in Walker (2007) [26].

Here, we propose a strategy that provides flexible approximations to the target predictive distribution. In addition, we look for models for which the required unsampled units can be easily simulated without any restriction on their values. Moreover, our proposal is able to cope with both the continuous and the discrete cases.

### 3.1. Continuous Variables

In the continuous case, we use a Gaussian Dirichlet process mixture DPM model for the unknown density function of the variable of interest in (2). Based on the experience with this model, a reasonable level of flexibility can be expected when using it to approximate continuous but otherwise arbitrary densities (see Escobar and West (1995) [28]). The analysis of this DPM model produces a posterior distribution over the set of continuous densities on the real line, and the predictive distribution for a single observation corresponds to the expectation of that posterior distribution. For our purposes, however, we need a sample of size *M*, say, from the joint posterior predictive distribution of the unsampled units. This is a multivariate distribution of dimension N−n whose components are not independent. Fortunately, this sample can be easily obtained if we independently simulate *M* densities from the posterior distribution mentioned above (each of these densities will be a *known* mixture of distributions) and then simulate (also *independently*) each unsampled unit from this univariate model. The slice sampler algorithm in Kalli, et al. (2011) [27] is used to follow these steps. See Appendix A for details concerning the MCMC algorithm used in this paper.

### 3.2. Discrete Variables

The case of discrete variables is, by far, more challenging from a parametric point of view. The use of Poisson and related models, as well as various mixtures thereof, has proved to be insufficient to describe the population distribution in a variety of applications. However, in a recent contribution, Canale and Dunson (2011) [29] developed a procedure to estimate the probability function of a discrete random variable that parallels that of Escobar and West (1995) [28]. Given a sample of a discrete random variable $\left\{X_{j}\right\}$, they introduce a collection of continuous latent variables $\left\{Y_{j}\right\}$ from which the discrete variables are obtained via a rounding process. The density of these latent variables f(y|G) is then modeled using a Gaussian DPM model as in (2), and the probability mass function p(x|G) of interest is obtained by integration over the rounding intervals which relate the latent variable and the discrete data, namely, $p\left(x=j|G\right)=\int_{a_{j}}^{a_{j+1}}f\left(y|G\right)dy,$ where $a_{j}$ and $a_{j+1}$ are thresholds lying on a suitable grid. Thus, this approach takes advantage of the flexibility of the continuous model in order to deal with the discrete case. The algorithm of Canale and Dunson (2011) [29] produces a nonparametric posterior distribution over the set of discrete distributions. As in the continuous case, we use this mechanism as a building block to simulate samples from the predictive distribution which corresponds to the expectation of this posterior distribution.

At any rate, after the simulation process we get *M* copies of the unsampled units on the population which, together with the units in the sample, provide *M* copies of the entire population. For each copy, the parameter of interest Q(X) can be computed, and thus a sample of size *M* from the predictive distribution of *Q* will be available for inference purposes. This procedure applies to any parameter, not just for the usual population mean or total. In the particular case of the total, it suffices to get the predictive distribution using only the unsampled units; the observed part can be later added as a constant. This is not the case for other nonlinear parameters such as the population quantiles. Nevertheless, the algorithm is general and can be used to produce inferences for any parameter. In the following section, several simulated examples as well as a real-data example are analyzed to show the type of results that can be obtained with this procedure when estimating the total, a quantile, and a ratio-type parameter of a finite population.

## 4. Implementation and Examples

As explained in the previous section, we propose to use a Bayesian nonparametric model as a prior for the distribution of θ in the population distribution p(x|θ). Here, we follow Escobar and West (1995) [28] and use a Dirichlet Process for this purpose. Specifically, we assume that, conditionally on G, the distribution of θ=(μ,V) follows a Dirichlet Process, where the base measure $G_{0}$ is given by a normal-inverse gamma distribution. The corresponding density is given by

$$g_{0}\left(\mu ,V|m,\tau ,s,S\right)=N\left(\mu |m,\tau V\right)\times \mathrm{InverseGamma}\left(V|s/2,S/2\right),$$

with hyperparameters m,τ,s and S, where τ is a positive scale factor and the Inverse-Gamma density has shape s/2 and scale S.

Let N(x|μ,V) denote a Normal distribution with mean μ and variance *V*. If p(x|θ)=N(x|μ,V), then *X* follows a Gaussian DPM. In order to obtain posterior samples via a Gibbs Sampler, we use a more recent algorithm than that of Escobar and West (1995) [28]. This algorithm is described in Kalli, et al. (2011) [27] and lies within the framework of slice sampler methods (e.g., Walker (2007) [26]. Because the Bayesian analysis for the Gaussian DPM produces a posterior distribution over the set of continuous densities on the real line, we can conceptualize posterior samples as realizations of a random continuous probability density. An important feature of the algorithms proposed by Walker (2007) [26] and Kalli, et al. (2011) [27] is that at each loop of the Gibbs sampler we have a posterior sample of this random density.

In the continuous case, once the convergence of the MCMC algorithm has been attained, at iteration i, the algorithm in Kalli, et al. (2011) [27] provides us with posterior samples $K_{i},\left(\pi_{1},\mu_{1},V_{1}\right),$$\ldots ,(\pi_{i},\mu_{K_{i}},V_{K_{i}}),$ which in turn define the posterior probability density

(3) $$f\left(x\right)=\pi_{1}N\left(x|\mu_{1},V_{1}\right)+\ldots +\pi_{K_{i}}N\left(x|\mu_{K_{i}},V_{K_{i}}\right).$$

Samples from this density can be used to approximate the predictive distribution of Q(X). With this in mind, we simulate the unobserved part of the population by (independently) sampling $X_{n+1},\ldots ,X_{N}$ from (3) and then, with these values and the observations $D=\{x_{1},\ldots ,x_{n}\}$, compute Q(X). We then repeat these steps for iterations j=i+1,…,i+M, to obtain a sample of size *M* from the predictive distribution of Q(X). In the case of a discrete variable, the simulated continuous variable is treated as latent and the observations of the variable of interest are obtained through the rounding process described in Section 3.2.

### 4.1. Simulated Examples

In each of the examples discussed in this section, we used the following values for the hyperparameters of the prior distribution: s=4, S=2, τ = 10,000, and $m={\bar{x}}_{n}$, where ${\bar{x}}_{n}$ is the sample mean.

#### 4.1.1. Continuous Case

**Figure 1:**
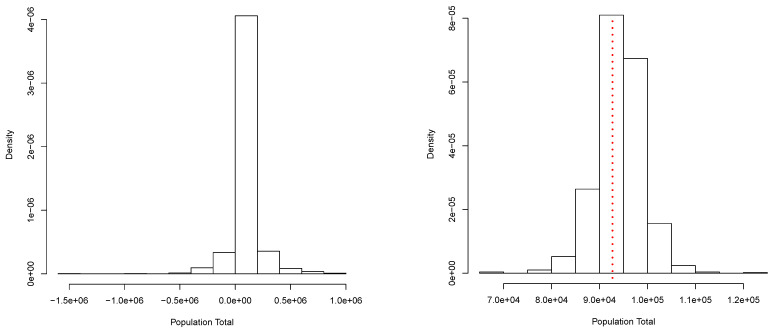
Prior (**left panel**) and posterior (**right panel**) predictive distribution of the population total. Continuous case.

A collection of N=6000 independent observations of a random variable *X* were generated according to the mixture f(x)=0.26N(x|10,9)+0.10N(x|7,4)+0.30N(x|13,16)+0.24N(x|22,6.25)+0.10N(x|30,16). This data set will be our finite population. Its total is *T* = 92,716.01. From this population, a random sample of size n=120 was obtained. From this sample, we get the posterior predictive distribution for Q(X)=T shown in Figure 1 (right panel). This corresponds to a simulation of M=1000 copies of the entire population, and the total *T* was computed for each copy. A Bayesian estimate for *T* can be produced once we choose an appropriate loss function. In particular, if we use a quadratic loss function, the estimate is given by E(T|D). In this case, we have $\hat{E(T|D)}$ = 94,202.64. Moreover, a 0.95 posterior predictive probability interval is given by (84,559.05, 103,520.17). These results can be compared with those obtained via the classical procedures: $\hat{T}$ = 93,914.97 and the (asymptotic) 95% confidence interval: (85836.7,101993.2). Figure 1 (right panel) shows the true total value *T* = 92,716.01 with a red dotted vertical line. Figure 1 (left panel) shows a simple approximation to the corresponding prior predictive distribution. This approximation was obtained using the same algorithm as for the posterior, but based on a minimal sample taken at random from the sample of size *n*. This shows how the sample contributes to the posterior predictive distribution of the parameter of interest.

#### 4.1.2. Discrete Case

**Figure 2:**
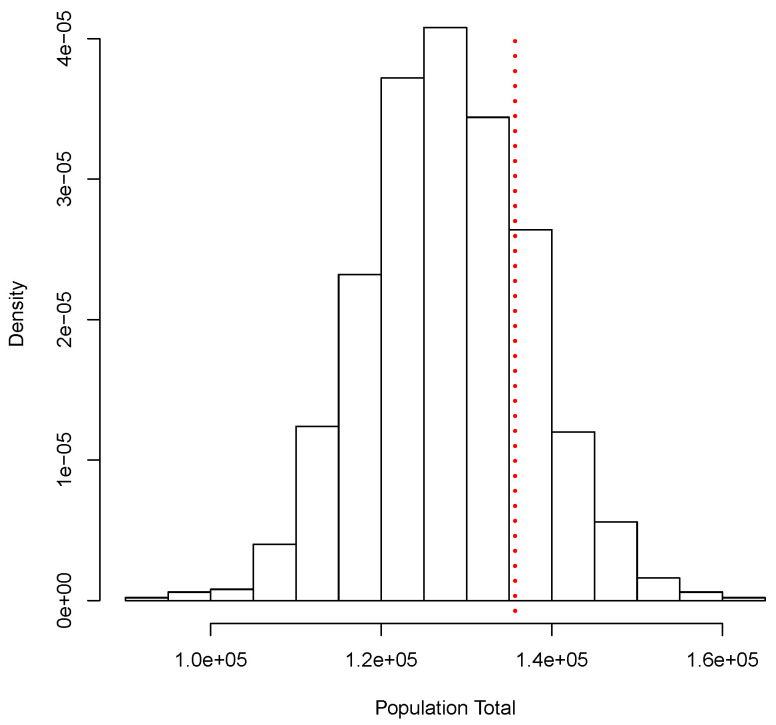
Posterior predictive distribution of the population total. Discrete case.

Similar to the continuous case, for this illustration we generated a set of N=6000 i.i.d. observations from the mixture f(x)=0.10Po(x|6)+0.15Po(x|14)+0.20Po(x|18)+0.10Po(x|22)+0.25Po(x|30)+0.20Po(x|32), where Po(x|λ) denotes the Poisson distribution with mean λ. This data set will take the role of our finite population. The corresponding total is given by *T* = 135,692. From this population, we obtained a random sample of size n=30 and used our proposal to estimate *T*. The posterior predictive distribution of *T* is shown in Figure 2. As before, this corresponds to a simulation of M=1000 copies of the entire population. Again, a Bayesian estimate for T, corresponding to a quadratic loss function is given by $\hat{E(T|D)}$ = 127,890.5, and a 0.95 posterior predictive probability interval is given by (109,463,146,872). The classical analysis leads to $\hat{T}$ = 128,200 with a 95% (asymptotic) confidence interval given by (107,047, 149,353). Figure 2 shows the true total value *T* = 135,692 with a red dotted vertical line. Both methods, Bayesian and classical, yield similar results.

### 4.2. A Real-Data Example

In many countries, the final results of the elections for president and governors are only released a few days after the election day. In order to mitigate the uncertainty that this delay may bring, both civil organizations and the media produce statistical estimates of the results, which are announced the very night of the election. In Mexico, the authority in charge of the electoral processes—the *Instituto Nacional Electoral* (INE)—produces its own estimates under the name of “INE Quick Count”.

To illustrate our proposal, we used a database with results for each one of the 7463 poll stations that the INE installed on 1 July 2018, for the Governor election in the State of Guanajuato (https://ieeg.mx/computos-finales/, accessed on 13 November 2020). A total number of 2,281,115 votes were recorded that day, and the election was won by Diego Sinhue Rodríguez Vallejo (DSRV) with 1,140,133 votes, 49.98% of the votes cast.

A random sample of 500 polling stations was chosen to produce our inferences. This is the same size the INE used for its Quick Count in 2018. There were other candidates in that election, but for the sake of simplicity, here we focus on the estimation of the proportion of votes obtained by DSRV. To this end, we defined two variables for each polling station: *X*, the number of votes in favor of DSRV, and *Y*, the total number of votes recorded. We need to estimate the population total of the variable *X* ($T^{X}$), the population total of the variable *Y* ($T^{Y}$), and the parameter of interest $P=T^{X}/T^{Y}$.

**Figure 3:**
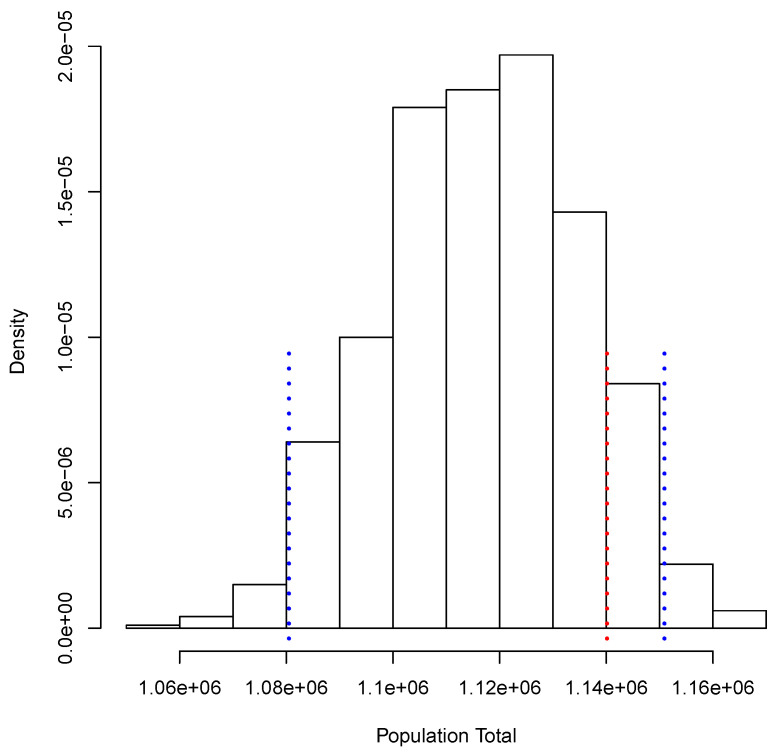
Posterior predictive distribution of the number of votes in favor of Diego Sinhue Rodríguez Vallejo (DSRV).

**Figure 4:**
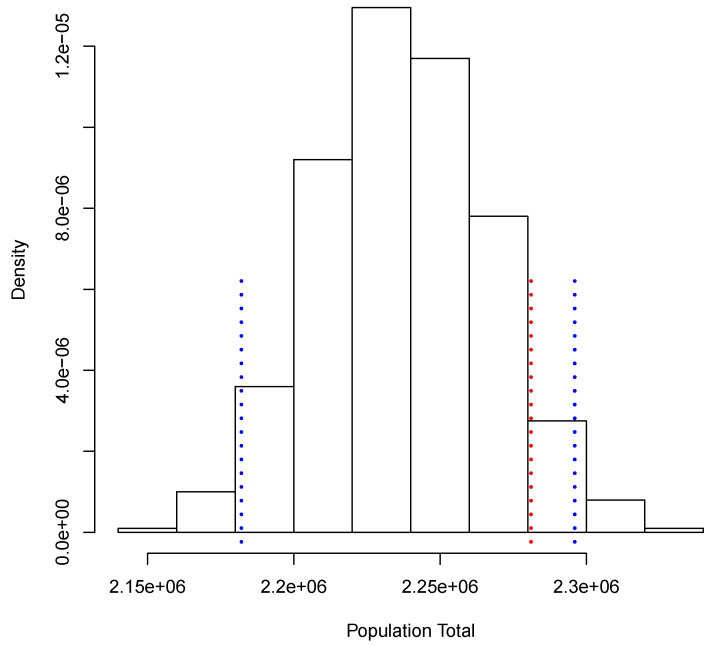
Posterior predictive distribution of the total number of votes recorded.

**Figure 5:**
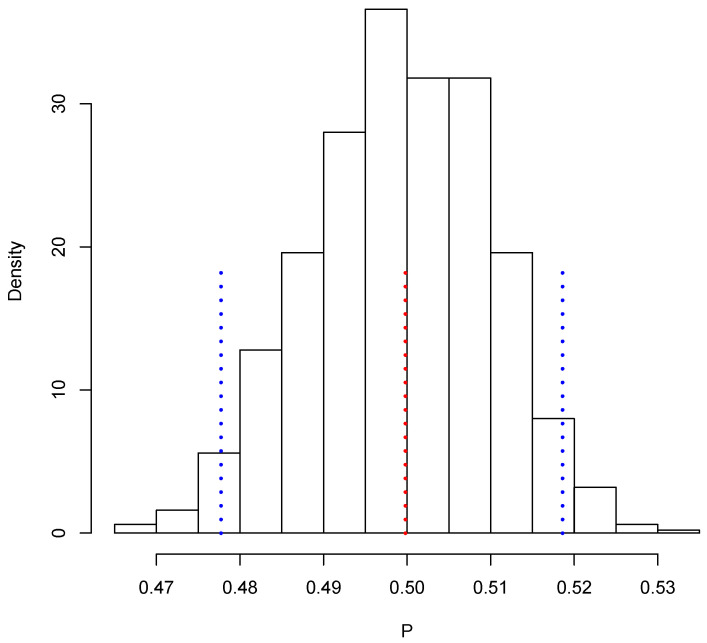
Posterior predictive distribution of *P*.

Figure 3 shows the posterior predictive distribution of $T^{X}$. We observe that this distribution is unimodal and asymmetric with a heavy tail on the right. The 0.95 probability interval is given by [1,080,506, 1,150,908], and it captures the true value 1,140,133. In the case of $T^{Y}$, the posterior predictive distribution is shown in Figure 4. This distribution is also unimodal and looks more symmetric; the 0.95 probability interval results as [2,182,122, 2,296,042]; it also captures the true value, 2,281,115. With the two simulated samples, $\left\{T_{i}^{X}:i=1,\ldots ,M\right\}$ and $\left\{T_{i}^{Y}:i=1,\ldots ,M\right\}$, we obtained another sample $\left\{P_{i}:i=1,\ldots ,M\right\}$ where $P_{i}=T_{i}^{X}/T_{i}^{Y}$ for i=1,…,M. The approximation to the posterior distribution of the parameter of interest *P* based on this sample appears in Figure 5. The distribution is unimodal and fairly symmetric, and the 0.95 probability interval is given by [0.4777,0.5186]. This interval captures rather well the true voting proportion, 0.4998. If these results were used to announce the outcome of the election the very night of the election day, the Quick Count would report that, with probability 0.95, the percentage of votes in favor of DSRV lies between 47.77% and 51.86% or, equivalently, that the percentage of votes in favor of DSRV lies in the interval 49.815%±2.045%.

### 4.3. Larger Population Sizes

To obtain an approximation which is close to the predictive distribution of Q(X), *M* may need to be in the range of thousands. On the other hand, in many real-life applications the population size *N* can be in units of tens or hundreds of thousands; in some cases, it could even reach millions. Thus, a loop implementing computations leading to the estimation of Q(X), as described after Equation (3), could easily require millions of iterations, making the procedure computationally unfeasible if results are required in a short period of time. Reducing the size of *M* will reduce the accuracy and precision of the approximation to the predictive density for Q(X). However, a more important issue is that the impact of taking smaller values of *M* may be insignificant if the value of *N* is sufficiently large. To get around this problem, we shall focus on a procedure to compute the simulated values of Q(X), which uses only a fraction of the unobserved part of the population. Specifically, in this section we will discuss how to take advantage of asymptotic theory in order to use only a rather small fraction of the unobserved population to approximate the value of Q(X).

For the sake of illustration, let us consider the case where the parameter Q(X) is the population total $Q\left(X\right)=\sum_{i=1}^{N}X_{i}$, and let

(4) $${\bar{X}}_{\left(N\right)}=Q\left(X\right)/N=\frac{1}{N}\sum_{i=1}^{N}X_{i},$$

be the population mean. This mean can be written as

$${\bar{X}}_{\left(N\right)}=\frac{1}{N}\left\{x_{1}+\ldots +x_{n}+\sum_{i=n+1}^{N}X_{i}\right\},$$

where the set $\left\{X_{n+1},\ldots ,X_{N}\right\}$ represents the unobserved part of the population. As described before, computing a predictive value of ${\bar{X}}_{\left(N\right)}$ can be very expensive, depending on the magnitude of *M* and, more dramatically, on the magnitude of N. Note that by defining ${\bar{x}}_{\left(n\right)}=\frac{1}{n}\sum_{i=1}^{n}x_{i}$ and ${\bar{X}}_{\left(N-n\right)}=\frac{1}{N-n}\sum_{j=1}^{N-n}X_{n+j},$ we can write

(5) $${\bar{X}}_{\left(N\right)}=\frac{1}{N}\left\{n\;{\bar{x}}_{\left(n\right)}+\left(N-n\right){\bar{X}}_{\left(N-n\right)}\right\}.$$

We propose to approximate ${\bar{X}}_{\left(N-n\right)}$ with ${\bar{X}}_{\left(R-n\right)}=\frac{1}{R-n}\sum_{j=1}^{R-n}X_{n+j},$ where *R* is an integer substantially smaller than N. Then, we will be using the approximation

$${\tilde{X}}_{N}=\frac{1}{N}\left\{n\;{\bar{x}}_{\left(n\right)}+\left(N-n\right){\bar{X}}_{\left(R-n\right)}\right\},$$

for the predictive value of ${\bar{X}}_{\left(N\right)}.$ This requires us to simulate R−n (instead of N−n) values from (3). As for the value of R, a simple argument based on the Central Limit Theorem can be used to propose a convenient value of *R* for the approximation.

For a large enough value of R, we have

$${\bar{X}}_{\left(R-n\right)}∼N\left({\bar{X}}_{\left(N\right)},\sigma^{2}/\left(R-n\right)\right),$$

where ${\bar{X}}_{\left(N\right)}$ is given by (4) and $\sigma^{2}=\mathrm{VAR}\left(X\right).$ These are the mean and the variance of the variable of interest *X* in the finite population of size N. Thus, for α∈(0,1),

$$P\left\{|{\bar{X}}_{\left(R-n\right)}-{\bar{X}}_{\left(N\right)}|\leq \frac{\sigma }{\sqrt{R-n}}Z_{\left(1-\alpha /2\right)}\right\}=1-\alpha$$

or, in terms of the relative error,

$$P\left\{\frac{|{\bar{X}}_{\left(R-n\right)}-{\bar{X}}_{\left(N\right)}|}{{\bar{X}}_{\left(N\right)}}\leq \left(\frac{\sigma }{{\bar{X}}_{\left(N\right)}}\right)\frac{Z_{\left(1-\alpha /2\right)}}{\sqrt{R-n}}\right\}=1-\alpha ,$$

where we assume ${\bar{X}}_{\left(N\right)}>0.$ Now assume this relative error is not larger than ϵ. Then, we must use *R* such that

$$\left(\frac{\sigma }{{\bar{X}}_{\left(N\right)}}\right)\frac{Z_{\left(1-\alpha /2\right)}}{\sqrt{R-n}}=\epsilon ,$$

leading to

$$R-n={\left(\frac{\sigma }{{\bar{X}}_{\left(N\right)}}\right)}^{2}{\left(\frac{Z_{\left(1-\alpha /2\right)}}{\epsilon }\right)}^{\;2}.$$

Finally, using the observed data, we can estimate ${\bar{X}}_{\left(N\right)}$ and $\sigma^{2}$ by ${\bar{x}}_{\left(n\right)}$ and $s_{\left(n\right)}^{2}=\frac{1}{n}\sum_{i=1}^{n}{\left(x_{i}-{\bar{x}}_{\left(n\right)}\right)}^{2}$, respectively, so that

$$R\approx n+{\left(\frac{Z_{\left(1-\alpha /2\right)}}{\epsilon }\right)}^{2}\times {\left(\frac{s_{\left(n\right)}}{{\bar{x}}_{\left(n\right)}}\right)}^{\;2}.$$

#### A Numerical Example

A population of size *N* = 100,000 was simulated from the model in Section 4.1.1, resulting in the population total of *T* = 1,548,577. From these simulated values we take a sample of n=1000 observations. For these data we have ${\bar{x}}_{\left(n\right)}=15.724,$$s_{\left(n\right)}=7.908.$ If, for example, we fix α=0.05 and ϵ=0.05, we get

$$\begin{array}{ccc} R & = & n+{\left(\frac{1.96}{0.05}\right)}^{2}\times {\left(\frac{7.908}{15.24}\right)}^{2}\\ & = & n+{\left(39.2\right)}^{2}\times {\left(0.5029663\right)}^{2}\\ & \approx & 1000+1537\times 0.252=1388. \end{array}$$

Thus, in this case we set R=1400. Following the argument of the previous section, once the convergence of the MCMC algorithm has been attained, at each loop we can simulate R−n=1400−1000=400 observations from the density (3) in order to approximate ${\bar{X}}_{\left(N-n\right)}$ in Equation (5). The reader may want to compare this with the task of simulating N−n= 99,000 observations at each loop, assuming that this procedure is repeated M=1000 times, say.

**Figure 6:**
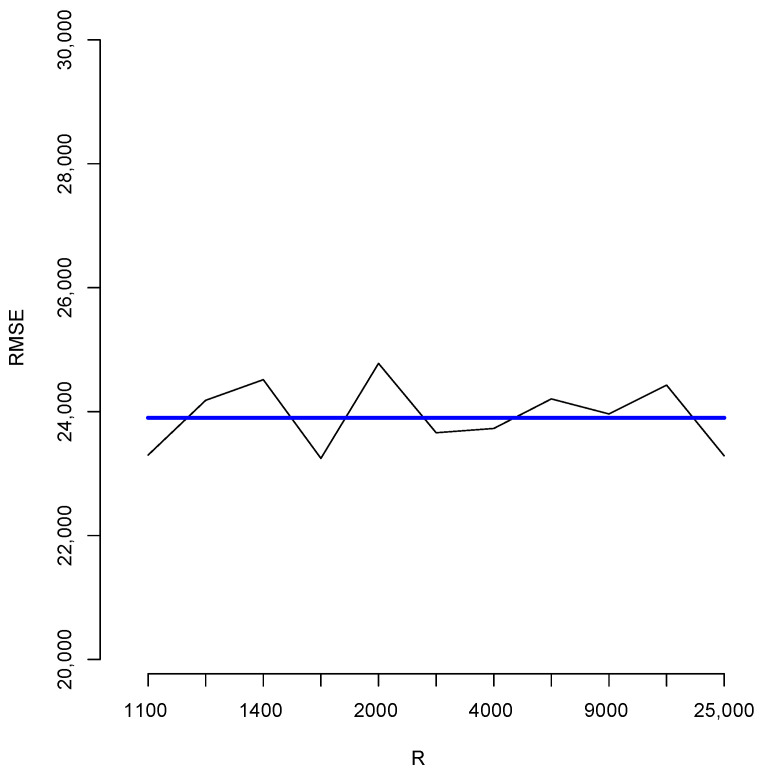
Root mean squared errors for different values of *R*.

**Figure 7:**
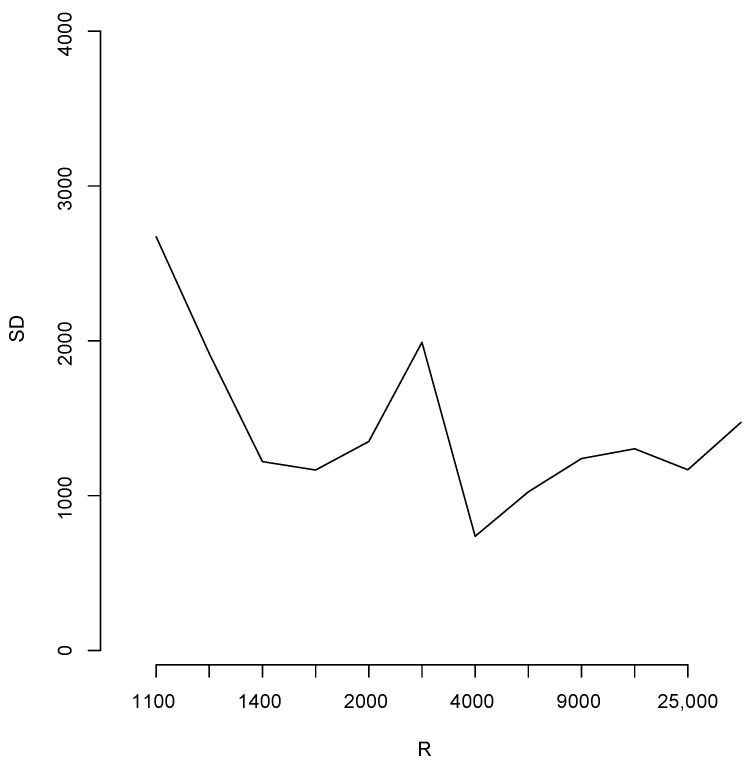
Sample standard deviation for different values of *R*.

**Figure 8:**
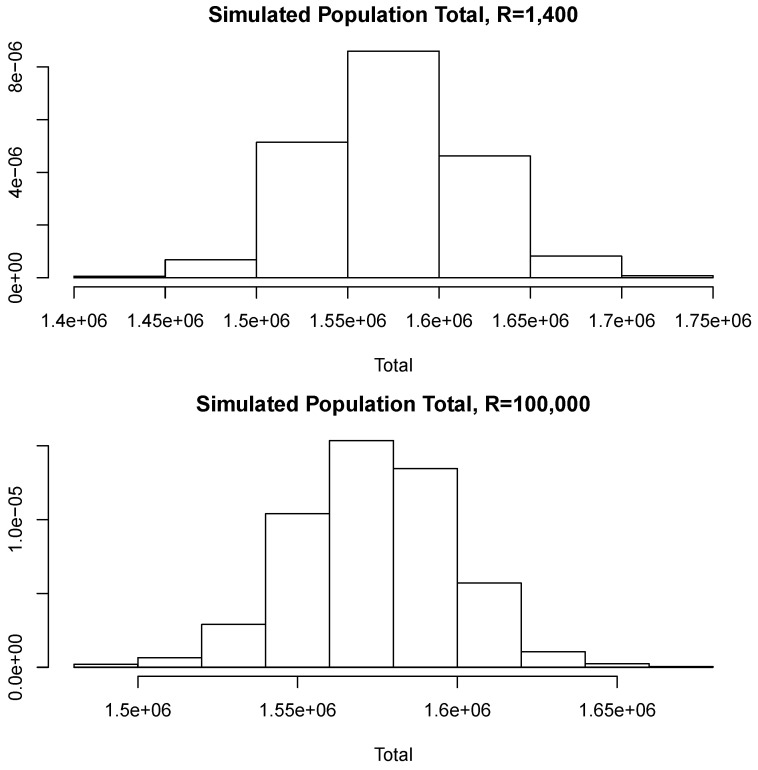
Simulated posterior predictive distributions.

With the aim of studying the effect of the magnitude of *R* in the estimates, we shall perform a brief simulation study using different values of *R*. To this end, for each fixed value of *R* we compute 10 different estimates of the population total *T*. Let us denote these by ${\hat{T}}_{1}^{\left(R\right)},\ldots ,{\hat{T}}_{10}^{\left(R\right)}$; Figure 6 reports the root mean squared error (RMSE), $\sqrt{\frac{1}{10}\sum_{t=1}^{10}{\left({\hat{T}}_{t}^{\left(R\right)}-T\right)}^{2}}$, for different values of *R* as indicated in the horizontal axis. The blue horizontal line corresponds to the value of the RMSE $\sqrt{\frac{1}{10}\sum_{t=1}^{10}{\left({\hat{T}}_{t}^{\left(100,000\right)}-T\right)}^{2}},$ obtained when R=N= 100,000, Note that, as all these RMSEs are not larger than 2500, then relative to the magnitude of the population total, the error in all cases is bounded by $\frac{25,000}{T}=0.016,$ i.e. 1.6%. Additionally, for each value of *R* in the horizontal axis, Figure 7 shows the sample standard deviation $\sqrt{\frac{1}{9}\sum_{t=1}^{10}{\left({\hat{T}}_{t}^{\left(R\right)}-{\bar{T}}_{\left(10\right)}\right)}^{2}}$ obtained from ${\hat{T}}_{1}^{\left(R\right)},\ldots ,{\hat{T}}_{10}^{\left(R\right)},$${\bar{T}}_{10}=\frac{1}{10}\sum_{t=1}^{10}{\hat{T}}_{t}^{\left(R\right)},$ again as a percentage of the population total. These deviations are bounded by $\frac{3000}{T}=0.0019\times 100\%,$ i.e., 0.19%. Figure 8 compares two simulated posterior predictive distributions, obtained using R=1400 (upper panel) and R= 100,000 (lower panel). As expected, the predictive distribution with the smaller value of *R* involves a larger amount of uncertainty. However, as the previous calculations show, this increase has a rather small relative effect on the value of the point-wise estimate. A similar conclusion is reached if the length of the probability intervals is also measured in relative terms.

**Figure 9:**
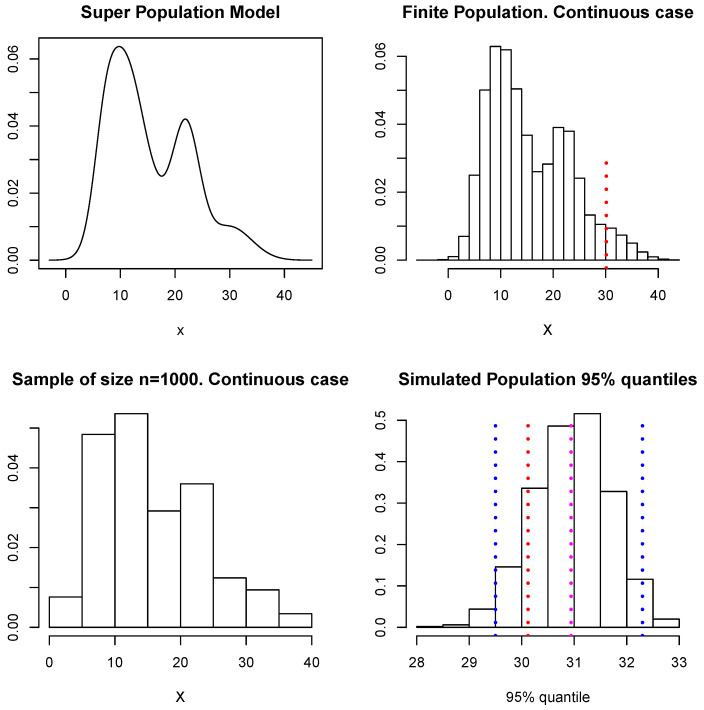
Superpopulation model, finite population, sample, and predictive distribution for the 95% quantile.

We shall now use these simulated data to illustrate the estimation of Q(X) given by the 95% quantile of the population values. Following the scheme described at the beginning of Section 4, at each loop of the Gibbs sampler we simulate the unobserved part of the population and use these values together with the sample *D* to compute the 95% quantile, so we end up with a sample of M=1000 simulated values of Q(X). The upper left panel in Figure 9 shows the model considered as the superpopulation model (this is described in Section 4.1.1). The upper right panel shows the finite population of N= 100,000 samples from the superpopulation model, there a (dotted) vertical red line shows the true 95% quantile of the finite population, which corresponds to a value of $X_{\left(0.95\right)}=30.12.$ The lower left panel shows the sample *D* of size n=1000. The lower right panel shows our approximation to the predictive distribution of the 95% quantile, and the blue (dotted) vertical lines show a 95% predictive interval for $X_{\left(0.95\right)}.$ This interval is given by (29.5,32.3) and contains the true value. Again, if a quadratic loss function is used, the point-wise estimate for $X_{\left(0.95\right)}$ is given by $\hat{E(Q(X)|D)}=30.94$; this is indicated in Figure 9 with a magenta dotted vertical line. In this example, all the unsampled values were simulated within the MCMC algorithm. In practice, however, a shortcut as that proposed for the means or totals can be explored to reduce the cost of the computation.

The approximate procedure to obtain inferences for the population mean and total is simple and feasible. With regard to other parameters, a similar asymptotic argument can be used to provide approximate inferences. In particular, in the case of quantiles, there are many contributions discussing the convergence of a sample quantile to its respective population counterpart. For example, the following result can be found in Section 2.3 of Serfling (1980) [30]. Let *X* be a random variable with density function f, for which the quantile of order *q* is given by $x_{\left(q\right)}$, and assume $f(x_{\left(q\right)})>0.$ Then, if $x_{\left(q:n\right)}$ is a sample quantile of order *q* from a random sample of size *n* of X, it follows that

$$\frac{n^{1/2}f\left(x_{\left(q\right)}\right)\left(x_{\left(q:n\right)}-x_{\left(q\right)}\right)}{{\left\{q\left(1-q\right)\right\}}^{1/2}}\;\;\mathrm{is}\;\;\mathrm{AN}\left(0,1\right)$$

where AN stands for “asymptotically normal”.

Thus, for *n* large enough

$$P\left(|x_{\left(q:n\right)}-x_{\left(q\right)}|\leq \frac{Z_{\left(1-\alpha /2\right)}{\left(q\left(1-q\right)\right)}^{1/2}}{n^{1/2}f\left(x_{\left(q\right)}\right)}\right)=1-\alpha .$$

If we fix the error bound

$$\frac{Z_{\left(1-\alpha /2\right)}{\left(q\left(1-q\right)\right)}^{1/2}}{n^{1/2}f\left(x_{\left(q\right)}\right)}=\epsilon$$

We get

$$n=\frac{Z_{\left(1-\alpha /2\right)}^{2}\left(q\left(1-q\right)\right)}{\epsilon^{2}{\left(f\left(x_{\left(q\right)}\right)\right)}^{2}}.$$

Here, all components are known with the exception of $f(x_{\left(q\right)}).$ However, in our case this quantity can be approximated with $\tilde{f}\left(x_{\left(q:n\right)}\right),$ where $\tilde{f}$ is the predictive density provided by the simulation process. Alternatively, we can use $\tilde{f}\left({\tilde{x}}_{\left(q\right)}\right),$ where ${\tilde{x}}_{\left(q\right)}$ is the quantile of order *q* corresponding to the predictive density $\tilde{f}.$ For other quantities of interest defined as functions of parameters for which asymptotic normality holds, the delta method could be used to provide the corresponding approximation rule.

**Figure 10:**
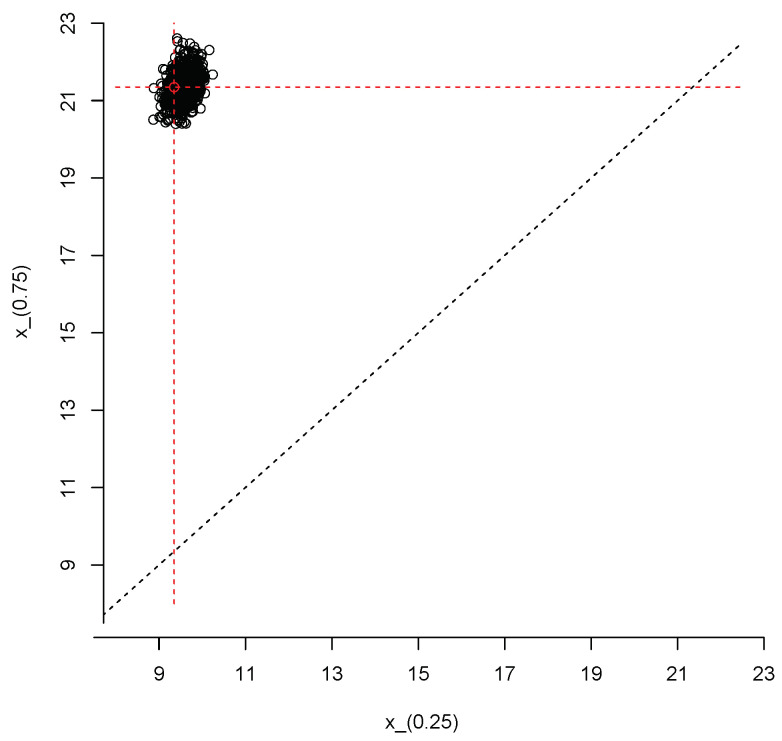
Simulated values from the joint posterior predictive distribution of $X_{\left(0.25\right)}$ and $X_{\left(0.75\right)}.$

**Figure 11:**
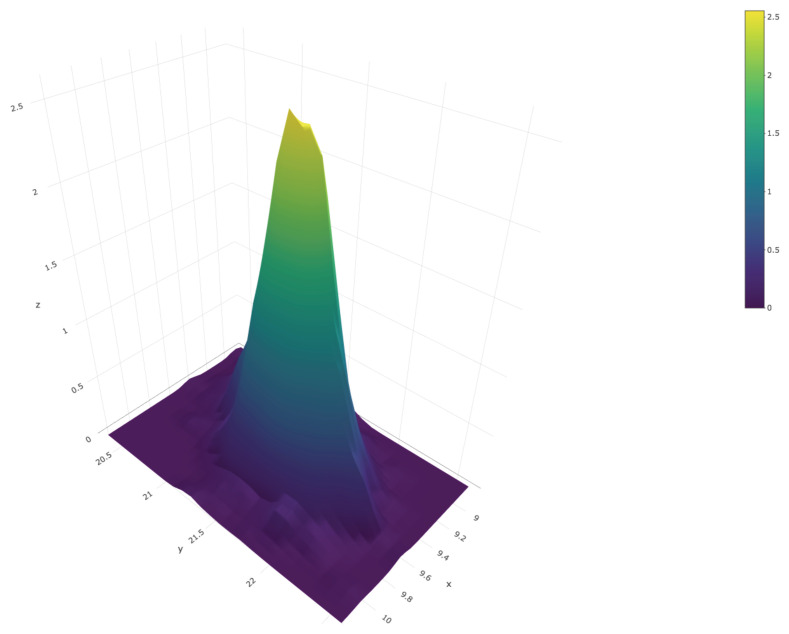
Joint posterior predictive density of $Q\left(X\right)=\left(X_{\left(0.25\right)},X_{\left(0.75\right)}\right).$

We close this section with an example that illustrates how to produce joint inferences about the first and third population quartiles, $X_{\left(0.25\right)}$ and $X_{\left(0.75\right)}$, respectively. We repeated the steps described after Equation (3) but, instead of the 95% quantile, at each iteration of the Gibbs sampler we computed $Q\left(X\right)=\left(X_{\left(0.25\right)},X_{\left(0.75\right)}\right)$. Thus, we end up with a sample of size M=1000 from the posterior predictive distribution of the vector Q(X). Figure 10 shows a scatter plot of this sample; the red dot represents the true values of the population quartiles, $X_{\left(0.25\right)}=9.35$ and $X_{\left(0.75\right)}=21.35$. The sample can also be used to produce an estimate of the joint density of $X_{\left(0.25\right)}$ and $X_{\left(0.75\right)}$; this is shown in Figure 11.

**Figure 12:**
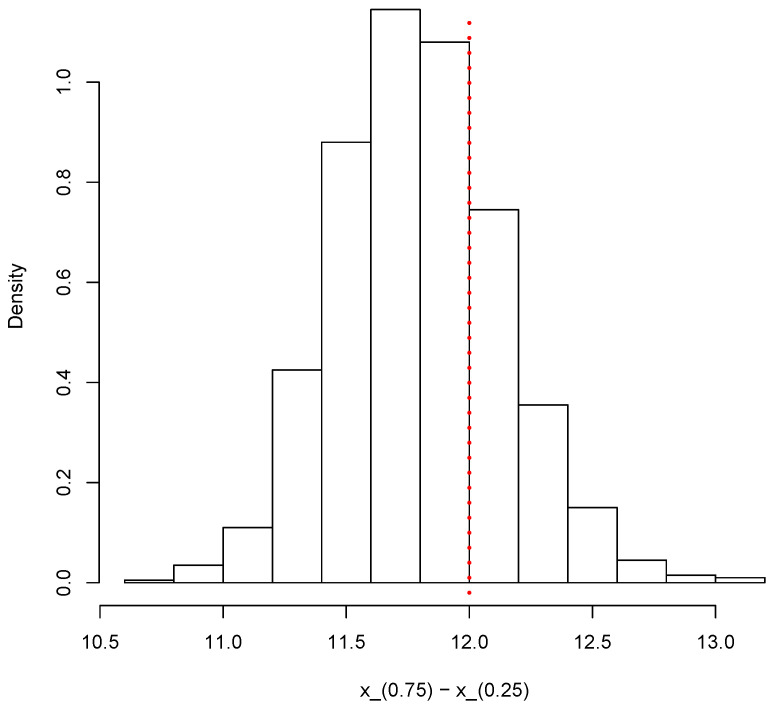
Posterior predictive distribution of the interquartile range $X_{\left(0.75\right)}-X_{\left(0.25\right)}.$

Finally, the same simulated sample can also be used to provide inferences on other relevant quantities such as the interquartile range $X_{\left(0.75\right)}-X_{\left(0.25\right)}.$ Figure 12 shows the posterior predictive distribution of the interquartile range. The vertical dotted red line shows the true value of the population interquartile range, which is 12 in this case.

## 5. Discussion

In recent years, a number of methods have been proposed to produce Bayesian inferences in the setting of survey sampling, with emphasis on the case of complex designs. One of the main differences among these contributions has to do with the way in which they incorporate the sampling weights into the analysis. Often, these procedures are nonparametric and rely on simulation techniques that render them uncompetitive, in terms of computing time, when compared with their frequentist counterparts. We would argue that there is no such thing as a standard Bayesian survey sampling method, even for the simpler designs. In this paper, we have focused on the simple random sampling case. The Bayesian survey sampling approach proposed here is nonparametric, and thus it avoids specific parametric assumptions that are often unwarranted. It can deal with both the continuous and the discrete cases in a unified manner, provides inferences for any parameter of the finite population of interest, and can be easily generalized to stratified sampling schemes. We propose that our approach be used as a kind of default procedure in the simple random sampling case. Moreover, we believe it can also be used as a benchmark for other proposals applicable to complex designs.

Admittedly, in its original formulation our approach can be as computationally expensive as the other methods. Fortunately, however, asymptotic results can be used to avoid the simulation of the entire unsampled part of the population. The modified procedure discussed in Section 4.3 is computationally efficient; this can be crucial in certain contexts, such as the analysis of quick counts in electoral settings. A limitation of this method, however, is that its applicability in the case of arbitrary parameters (other than the population mean, total, or quantiles) depends on the availability of suitable asymptotic results.

A proper comparison would involve a more thorough simulation exercise under a wide range of scenarios. Nevertheless, note that even in this limited first study, our proposal produces competitive results and provides the user with a set of samples from the predictive distribution of X that can be used to estimate any characteristic of interest in the population under study. An important instance arises when we are interested in a population quantile instead of the total of an attribute in the population. In such a case, commonly used frequentist procedures no longer provide a simple, automatic way to produce interval estimates except in the asymptotic scenario.

## 6. Materials and Methods

All of the examples of Section 4 were implemented using the R statistical language and environment (R Core Team, 2020 [31]).

## Data Availability

The dataset analyzed in Section 4.2 is publicly available at: https://ieeg.mx/computos-finales/ (accessed on 13 November 2020).

## Abbreviations

The following abbreviations are used in this manuscript:

DPM	Dirichlet Process Mixture
DSRV	Diego Sinhue Rodríguez Vallejo
INE	Instituto Nacional Electoral
MCMC	Markov Chain Monte Carlo

## Appendix A

Consider the Sethuraman [25] representation for the Dirichlet process given in Section 3 which leads to Equation (2), where f(x|θ)=N(x|μ,V). For the data $\{x_{1},\ldots ,x_{n}\},$ Walker (2007) [26] and Kalli, et al. (2011) [27] introduce latent variables $\left\{u_{i}:i=1,\ldots ,n\right\}$ and $\{d_{i}:i=1,\ldots ,n\},$ such that $0<u_{i}<1,$ i=1,…,n and $d_{i}$ is a finite integer, i=1,…,n. Walker (2007) [26] states that the vector $\left(x_{i},u_{i},d_{i}\right)$ has joint density given by

$$f\left(x_{i},u_{i},d_{i}=k|G\right)=1\left(u_{i}<\omega_{k}\right)N\left(x_{i}|\mu_{k},V_{k}\right),$$

with corresponding marginal $f(x_{i}|G)$ given in (2). For each i, the role of $u_{i}$ is to simplify (2) to a finite sum, with a number of terms given by the cardinality $N_{i}$ of the set $A\left(u_{i}\right)=\left\{j:u_{i}<\omega_{j}\right\},$ on the other hand, $d_{i}$ is a selector, whose value indicates which term in this finite sum generated the observation $x_{i}.$ A usual problem with slice sampler algorithms, is that posterior samples of the latent (augmented) variables feature high correlation implying slow mixing. Additionally, for the method proposed in Walker (2007) [26], when sampling $u_{1},...,u_{n},$ the cardinality of the sets $A(u_{i})$ may increase, making numerical computations slow. To overcome these problems, Kalli, et al. (2011) [27] suggest using a deterministic and decreasing sequence ${\left\{\xi_{j}\right\}}_{j=1}^{\infty },$ such that $\xi_{j}>0,$j=1,2,…, in order to define the joint density

(A1) $$f\left(x_{i},u_{i},d_{i}=k|G\right)=1\left(u_{i}<\xi_{k}\right)\left(\frac{\omega_{k}}{\xi_{k}}\right)N\left(x_{i}|\mu_{k},V_{k}\right),$$

which also has corresponding marginal for $x_{i}$ given by (2). For our implementation, we used one of the possibilities suggested by Kalli, et al. (2011) [27] for the sequence ${\left\{\xi_{j}\right\}}_{j=1}^{\infty },$ namely, $\xi_{j}={\left\{\frac{1}{2}\right\}}^{j},$j=1,2,….

For each i=1,…,n, let us denote by $N_{i}$ the cardinality of the finite set $\{j:\xi_{j}>u_{i}\},$ the selector $d_{i}$ takes values in the set $1,2,\ldots ,N_{i}.$ Kalli, et al. (2011) [27] state that the augmented likelihood corresponding to the a sample of size n,${\left\{x_{i},u_{i},d_{i}\right\}}_{i=1}^{n}$ is

(A2) $$l\left({\left\{x_{i},u_{i},d_{i}\right\}}_{i=1}^{n}|G\right)=\prod_{i=1}^{n}1\left(u_{i}<\xi_{d_{i}}\right)\left(\frac{w_{d_{i}}}{\xi_{d_{i}}}\right)N\left(x_{i}|\mu_{d_{i}},V_{d_{i}}\right).$$

Let us denote by $g_{0}\left(\mu ,V|m,\tau ,s,S\right)$ the Normal-Inverse gamma density with hyperparameters m,τ,s,S, the prior distribution for the sequences ${\left\{\theta_{j}=\left(\mu_{j},V_{j}\right)\right\}}_{j}$ and ${\left\{\upsilon_{j}\right\}}_{j}$ in Sethuraman representation for a Dirichlet Process is given by

(a) ${\left\{\left(\mu_{j},V_{j}\right)\right\}}_{j=1}^{\infty }$ is a sequence of independent random variables from $g_{0}\left(\mu ,V|m\tau ,s,S\right).$
(b) ${\left\{\upsilon_{j}\right\}}_{j=1}^{\infty }$ are independent and with distribution beta(υ|1,α).

The augmented likelihood (A2) and the prior for the sequences ${\left\{\left(\mu_{j},V_{j}\right)\right\}}_{j}$ and ${\left\{\upsilon_{j}\right\}}_{j}$ given in (a) and (b) can be used with Bayes’ theorem to obtain the full conditional distributions for all unknown quantities. Note that as the introduction of the latent variable $u_{i}$ yields a finite sum and the selector $d_{i}$ takes values in the set $\{1,2,...,N_{i}\},$ then at each loop of the Gibbs sampler, samples of the parameters $\left(\mu_{j},V_{j}\right)$ and $\upsilon_{j}$ are required only for *j* in the set {1,…,N}, where $N=\max\{N_{1},\ldots ,N_{n}\}.$ Let $D_{j}$ denote the set $\left\{i:d_{i}=j\right\}$ and $n_{j}$ be the cardinality of $D_{j},$ the full conditional distributions for the Gibbs sampler are

(fc1) $\pi \left(\mu_{j}|\cdots \right)=N\left(\mu_{j}\left|\frac{\frac{m}{\tau }+\sum_{i\in D_{j}}x_{i}}{\frac{1}{\tau }+n_{j}};\frac{\tau V_{j}}{1+\tau n_{j}}\right.\right).$
(fc2) $\pi \left(V_{j}|\cdots \right)=\mathrm{InverseGamma}\left(V_{j}\left|\frac{n_{j}+s+1}{2};\frac{{\left(\mu_{j}-m\right)}^{2}}{2\tau }+\frac{S}{2}+\frac{\sum_{i\in D_{j}}{\left(x_{i}-\mu_{j}\right)}^{2}}{2}\right.\right).$
(fc3) $\pi \left(\upsilon_{j}|\cdots \right)=\mathrm{Beta}\left(\upsilon_{j}|1+n_{j};\alpha +\sum_{i=1}^{n}1\left(d_{i}>j\right)\right).$
(fc4) $\pi \left(u_{i}|\cdots \right)\propto 1\left(0<u_{i}<\xi_{d_{i}}\right).$
(fc5) $\pi \left(d_{i}=k|\cdots \right)\propto 1\left(k:u_{i}<\xi_{k}\right)\;\frac{w_{k}}{\xi_{k}}\;N\left(x_{i}|\mu_{k},V_{k}\right).$

We use the next algorithm to implement a Gibbs sampler based on the full conditional distributions above.

### Algorithm

- At the *t*-th step, assume we have sampled values
  $${\left\{u_{i,t-1}\right\}}_{i=1}^{n};\;{\left\{d_{i,t-1}\right\}}_{i=1}^{n};\;{\left\{\left(\mu_{j,t-1},V_{j,t-1}\right)\right\}}_{j=1}^{N^{\left(t-1\right)}};\;{\left\{\upsilon_{j,t-1}\right\}}_{j=1}^{N^{\left(t-1\right)}},$$
  where the number of components in the mixture is $N^{\left(t-1\right)}.$
- Begin by obtaining samples $u_{1,t},\ldots ,u_{n,t},$ where each $u_{i,t}$ is sampled from a $\mathrm{Uniform}(u|0,\xi_{d_{i,t-1}})$ density, i=1,…,n. Use these to obtain $N_{i}=\max\left\{l:\xi_{l}>u_{i,t}\right\},$i=1,…,n, then update the *number of components*$N^{\left(t\right)}=\max\left\{N_{1},\ldots ,N_{n}\right\}.$
- To obtain ${\left\{\left(\mu_{j,t},V_{j,t}\right)\right\}}_{j=1}^{N^{\left(t\right)}};\;{\left\{\upsilon_{j,t}\right\}}_{j=1}^{N^{\left(t\right)}}$ consider two cases:
  (i) If $N^{\left(t\right)}<=N^{\left(t-1\right)},$ then for each $j=1,2,\ldots ,N^{\left(t\right)}$
    (i.1) if $n_{j}>0,$ then replace $\left(\mu_{j,t-1},V_{j,t-1}\right)$ by a sample $\left(\mu_{j,t},V_{j,t}\right)$ from (fc1) and (fc2) above.
    (i.2) if $n_{j}=0,$ then replace $\left(\mu_{j,t-1},V_{j,t-1}\right)$ by a sample $\left(\mu_{j,t},V_{j,t}\right)$ from $g_{0}\left(\mu ,V|m,\tau ,s,S\right).$
    Then, obtain a sample $\upsilon_{j,t}$ from (fc3). These steps define the updated set of parameters ${\left\{\left(\mu_{j,t},V_{j,t}\right)\right\}}_{j=1}^{N^{\left(t\right)}};\;{\left\{\upsilon_{j,t}\right\}}_{j=1}^{N^{\left(t\right)}}.$
  (ii) if $N^{\left(t\right)}>N^{\left(t-1\right)},$ then for each $j=1,2,\ldots ,N^{\left(t-1\right)}$ produce a sample $\left(\mu_{j,t},V_{j,t}\right)$ by repeating steps (i.1) or (i.2) depending on the value of $n_{j}.$ For each $j=N^{\left(t-1\right)}+1,\ldots ,N^{\left(t\right)},$ produce a sample $\left(\mu_{j,t},V_{j,t}\right)$ from $g_{0}\left(\mu ,V|m,\tau ,s,S\right).$ Then, for each $j=1,2,\ldots ,N^{\left(t\right)}$ obtain a sample $\upsilon_{j,t}$ from (fc3). These steps define the updated set of parameters ${\left\{\left(\mu_{j,t},V_{j,t}\right)\right\}}_{j=1}^{N^{\left(t\right)}};\;{\left\{\upsilon_{j,t}\right\}}_{j=1}^{N^{\left(t\right)}}.$
- Use ${\left\{\upsilon_{j,t}\right\}}_{j=1}^{N^{\left(t\right)}}$ to compute ${\left\{w_{j,t}\right\}}_{j=1}^{N^{\left(t\right)}}$ by using the relations $w_{1}=\upsilon_{1},$$w_{j}=\upsilon_{j}\prod_{l<j}\left(1-\upsilon_{l}\right),$ then update the selectors by sampling ${\left\{d_{i,t}\right\}}_{i=1}^{n}$ from the full conditional (fc5).
